# Pharmacological inhibition of MALT1 protease activity protects mice in a mouse model of multiple sclerosis

**DOI:** 10.1186/1742-2094-11-124

**Published:** 2014-07-21

**Authors:** Conor Mc Guire, Lynn Elton, Peter Wieghofer, Jens Staal, Sofie Voet, Annelies Demeyer, Daniel Nagel, Daniel Krappmann, Marco Prinz, Rudi Beyaert, Geert van Loo

**Affiliations:** 1 Inflammation Research Center, Unit of Molecular Signal Transduction in Inflammation, VIB, Technologiepark 927, B-9052 Ghent, Belgium; 2 Department of Biomedical Molecular Biology, Ghent University, Technologiepark 927, B-9052 Ghent, Belgium; 3 Department of Neuropathology and Faculty of Biology, University of Freiburg, Breisacherstrasse 64, D-79106 Freiburg, Germany; 4 BIOSS Centre for Biological Signaling Studies, University of Freiburg, Breisacherstrasse 64, D79106 Freiburg, Germany; 5 Institute for Molecular Toxicology and Pharmacology, Cellular Signal Integration, Helmholtz Zentrum München - German Research Center for Environmental Health, Ingolstädter Landstr. 1, D-85764 Neuherberg, Germany

**Keywords:** multiple sclerosis, experimental autoimmune encephalomyelitis, MALT1, mepazine, demyelination

## Abstract

**Background:**

The paracaspase mucosa-associated lymphoid tissue lymphoma translocation protein 1 (MALT1) is crucial for lymphocyte activation through signaling to the transcription factor NF-κB. Besides functioning as a scaffold signaling protein, MALT1 also acts as a cysteine protease that specifically cleaves a number of substrates and contributes to specific T cell receptor-induced gene expression. Recently, small molecule inhibitors of MALT1 proteolytic activity were identified and shown to have promising anticancer properties in subtypes of B cell lymphoma. However, information on the therapeutic potential of small compound inhibitors that target MALT1 protease activity in autoimmunity is still lacking.

**Methods:**

The present study aimed to elucidate whether MALT1 protease inhibitors are also useful in the treatment of lymphocyte-mediated autoimmune pathologies such as multiple sclerosis (MS). For this, we studied the therapeutic potential of a recently identified inhibitor of MALT1 protease activity, the phenothiazine derivative mepazine, in the context of experimental autoimmune encephalomyelitis (EAE), the main animal model for MS.

**Results:**

We demonstrate that administration of mepazine prophylactically or after disease onset, can attenuate EAE. Importantly, while complete absence of MALT1 affects the differentiation of regulatory T (Treg) cells *in vivo*, the MALT1 protease inhibitor mepazine did not affect Treg development.

**Conclusions:**

Altogether, these data indicate that small molecule inhibitors of MALT1 not only hold great promise for the treatment of B cell lymphomas but also for autoimmune disorders such as MS.

## Background

Multiple sclerosis (MS) is the most common chronic inflammatory demyelinating disease of the central nervous system (CNS) [1]. MS and experimental autoimmune encephalomyelitis (EAE), its main animal model, are characterized by myelin-specific autoreactive T cells, which home to the CNS where they initiate a vicious cycle of inflammation and tissue damage, eventually leading to oligodendrocyte cell death and subsequent axonal demyelination and degeneration [2]. The transcription factor NF-κB plays a central role in T cell activation and proliferation. Upon stimulation of the T cell receptor (TCR), protein kinase C θ-mediated phosphorylation of CARMA1 results in the recruitment of the scaffolding proteins Bcl-10 and MALT1, forming the so-called CBM complex, which mediates the recruitment of the E3 ubiquitin ligase TRAF6 and other downstream signaling molecules that are essential for the activation of the IκB kinase (IKK) complex. Next to its scaffold function, MALT1 has recently been shown to also have enzymatic activity [3-7]. In contrast to MALT1’s scaffolding function, its protease activity is not crucial for IKK activation [8], but supports specific TCR-induced gene expression by inactivating negative regulators such as the de-ubiquitinases A20 [3] and CYLD [6], the NF-κB family member RelB [4], and the RNase Regnase-1 [7].

At the cellular level, MALT1 proteolytic activity has been implicated in MALT lymphoma and activated B cell-like diffuse large B cell lymphoma (ABC-DLBCL) [3,9-11]. In this context, treatment of ABC-DLBCL cells with the MALT1 protease peptide inhibitor z-VRPR-fmk reduces constitutive NF-κB activity and cell proliferation [12,13]. Recently, small molecule MALT1 protease inhibitors also were described and shown to suppress proliferation of ABC-DLBCL *in vitro* and in xenotransplanted tumors *in vivo*[14,15], positioning MALT1 as an attractive anticancer drug target. The phenothiazine derivative mepazine was shown to act as a potent noncompetitive MALT1 inhibitor by binding to an allosteric pocket on MALT1 [16]. The reversible mode of action and effectiveness of MALT1 inhibition by mepazine *in vivo *[15] indicates a possible clinical use also for the treatment of severe autoimmune diseases. Therefore, we here assessed the potential of mepazine on the development and progression of EAE. We demonstrate significant protective activities of mepazine when administered either early (before the onset of acute disease) or late (at the peak of disease), illustrating that MALT1 is not only an attractive drug target in cancer but also in autoimmunity.

## Methods

### Mice

Male mice aged 8 to 10 weeks old were purchased from Janvier N.V. and kept in an specific pathogen free (SPF) animal facility in individually ventilated cages. Food and water was provided *ad libitum* for the duration of the experiments. All animal experiments were performed according to institutional, national and European guidelines and were approved by the Gent University institutional review committee.

### Experimental autoimmune encephalomyelitis induction and follow-up

EAE was induced as previously described [17]. Briefly, mice were subcutaneously immunized with an emulsion of 200 μg myelin oligodendrocyte glycoprotein peptide (MOG_35-55_) peptide (Charite, Institute for Medical Immunology, Berlin, Germany) and complete Freund’s adjuvant (Sigma-Aldrich, St. Louis, MO, USA) supplemented with 10 mg/ml Mycobacterium Tuberculosis H37RA (BD Bioscience, San Diego, CA, USA). On the day of immunization and 48 hours after, mice also received 50 ng of pertussis toxin (Sigma-Aldrich, St. Louis, MO, USA) in sterile PBS intraperitoneally (i.p.). For passive induction of EAE, spleens from immunized mice were isolated 10 days post-immunization. Splenocytes were cultured in RPMI 1640 supplemented with 10% FBS, sodium pyruvate, L-glutamine, nonessential amino acids, antibiotics, 30 μM MOG_35-55_ peptide and 10 ng/ml recombinant mouse IL-23 (eBioscience, San Diego, CA, USA). After 48 hours, splenocytes were harvested, washed and resuspended in PBS. A total of 3 × 10^7^ cells were injected intravenously (i.v.) into recipient mice, which were sub-lethally irradiated (400 cGy) 24 hours prior to cell transfer. Mice received 200 ng pertussis toxin i.p. on the day of cell transfer and 48 hours later. Body weight and clinical disease development were followed up daily. Paralysis was scored according to a scale as follows: 0, normal; 1, weakness of tail; 2, complete loss of tail tonicity; 3, partial hind limb paralysis; 4, complete hind limb paralysis; 5, forelimb paralysis or moribund; and 6, death. Intermediate scores of 0.5 were given when necessary. To eliminate any diagnostic bias, mice were scored blindly.

### Treatment with mepazine

Mice were randomly treated with either mepazine or control solution to eliminate possible cage effects. Mepazine acetate (Chembridge, San Diego, CA, USA) was solubilized in 0.7 × PBS at a concentration of 2 mg/ml. Mice were injected i.p. twice daily with 8 mg/kg starting at day 7 post-immunization, or between days 14 and 17 when they reached a clinical score of 2. For adoptive transfer EAE, donor mice were injected with mepazine or vehicle from the day of immunization until the isolation of splenocytes.

### Histological analysis

Mice were transcardially perfused with PBS containing 5 IU/ml heparin (De Pannemaeker N.V., Gent, Belgium) followed by perfusion with 4% paraformaldehyde. Spinal cords were dissected, dehydrated and embedded in paraffin blocks. Sections of 2 μm were stained with hematoxylin and eosin (H & E), Luxol fast blue (LFB) (Solvent Blue 38, practical grade, Sigma Genosys, The Woodlands, TX, USA) for assessment of demyelination, and antibodies against CD3 (Clone CD3-12, Serotec, Raleigh, NC, USA), Mac-3 (Clone CD107b, M3/84, BD Biosciences, San Diego, CA, USA), B220 (Clone RA3-6B2, BD Biosciences, San Diego, CA, USA) or amyloid precursor protein (APP) (Clone 22C11, Millipore, Darmstadt, Germany). Sections were rehydrated and incubated in 10 mM citrate buffer for 5 minutes at 94°C. Nonspecific binding was blocked by incubating sections in 0.1 M PBS containing 10% FCS and 1% Triton x-100 for 30 minutes. Primary antibodies were incubated overnight at 4°C. Histological quantification was described previously [18].

### Cytokine analysis and quantitative real-time PCR

Total RNA was isolated using TRIzol reagent (Invitrogen) and Aurum Total RNA Isolation Mini Kit (Bio-Rad Laboratories, Hercules, CA, USA) according to manufacturer’s instructions. Synthesis of cDNA was performed using iScript cDNA synthesis kit (Bio-Rad Laboratories, Hercules, CA, USA) according to the manufacturer’s instructions. A total of 10 ng of cDNA was used for quantitative PCR in a total volume of 10 μl with SensiFast SYBR No-Rox Kit (GeC Biotech, London, UK) and specific primers on a LightCycler 480 (Roche). Real-time PCR reactions were performed in triplicates. The following mouse-specific primers were used: hprt forward, 5’-AGTGTTGGATACAGGCCAGAC-3’; hprt reverse, 5’-CCGTGATTCAAATCCCTGAAGT-3’; gapdh forward, 5’-TGAAGCAGGCATCTGAGGG-3’; gapdh reverse, 5’-CGAAGGTGGAAGAGTGGGAG-3’; IFNγ forward, 5’-GCCAAGCGGCTGACTGA-3’; IFNγ reverse, 5’-TCAGTGAAGTAAAGGTACAAGCTACAATCT-3’; IL-2 forward, 5’-GTGCCAATTCGATGATGAGTCA-3’; IL-2 reverse, 5’-GGGCTTGTTGAGATGATGCTTT-3’; tbet forward, 5’- AGAACGCAGAGATCACTCAG-3’; tbet reverse, 5’- GGATACTGGTTGGATAGAAGAGG-3’; IL-13 forward, 5’-TCAGCCATGAAATAACTTATTGTTTTGT-3’; IL-13 reverse, 5’-CCTTGAGTGTAACAGGCCATTCT-3’; STAT6 forward, 5’-GGGTGTTAATGCTCGAATGTGATA-3’; STAT6 reverse 5’-CACAATGTCTCTATGTTTCTG TATGTTGAG-3’; GATA3 forward, 5’-GGCAGAAAGCAAAATGTTTGCT-3’; GATA3 reverse, 5’-TGAGTCTGAATGGCTTATTCACAAAT-3’; IL-6 forward, 5’-GAGGATACCACTCCCAACAGACC-3’; IL-6 reverse, 5’-AAGTGCATCATCGTTGTTCATACA-3’; RORγt forward, 5’-TGTCCTGGGCTACCCTACT-3’; RORγt reverse, 5’-GCACCCCTCACAGGTGATAA-3’; IL-17A forward, 5’-CAGGACGCGCAAACATGA-3’; IL-17A reverse, 5’-GCAACAGCATCAGAGACACAGAT-3’; Foxp3 forward, 5’-TTCCTTCCCAGAGTTCTTCC-3’; Foxp3 reverse, 5’-CTCAAATTCATCTACGGTCCAC -3’; IL-10 forward, 5’-GGTGTCCTTTCAATTGCTCTCAT -3’; IL-10 reverse, 5’-TCACAACTCTCTTAGGAGCTCTGAACT -3’; TGFβ forward, 5’-GCTGAACCAAGGAGACGGAATA -3’ and TGFβ reverse, 5’-GAGTTTGTTATCTTTGCTGTCACAAGA -3’.

### T cell recall assay

T cell recall responses were assessed in splenocytes isolated from 10 d post-immunization with MOG_35-55_ peptide. Erythrocytes were lysed using ACK lysis buffer and splenocytes were cultured in flat-bottomed 96-well plates at a density of 7 × 10^5^ cells/well in DMEM supplemented with 5% FCS, L-glutamine, nonessential amino acids and antibiotics. Cells were pretreated with 13 μM mepazine for 1 hour, after which 1, 10 or 30 μg/ml MOG_35-55_ peptide was added. After 48 hours, supernatant was collected and concentrations of IL-2, IFNγ and IL-17 were determined by ELISA (eBioscience, San Diego, CA, USA).

### Cell culture and immunoblot

CD4^+^ T cells were purified from naïve mice using the CD4^+^ T cell isolation kit (Miltenyi Biotech, Bergisch Gladbach, Germany) according to the manufacturer’s instructions. T cells were cultured in RPMI 1640 supplemented with 10% fetal bovine serum, L-glutamine, sodium pyruvate, non-essential amino acids and antibiotics. Jurkat cells were cultured as described before [6]. Cells were pretreated with mepazine, after which they were stimulated with 200 ng/ml PMA (Sigma-Aldrich, , St. Louis, MO, USA) and 1 μM ionomycin (Calbiochem, Darmstadt, Germany) in the presence or absence of mepazine for indicated time points. After stimulation, cells were lysed in 50 mM Hepes, pH 7.6, 250 mM NaCl, 5 mM EDTA, 0.5% NP-40 and phosphatase and protease inhibitors. Proteins were separated by SDS-PAGE and analyzed by semi-dry immunoblotting and detection via enhanced chemiluminescence (Perkin-Elmer Life Sciences, Waltham, MA, USA). Antibodies that were used are anti-A20 (clone 59A426, eBioscience, San Diego, CA, USA), anti-CYLD (E10, Santa Cruz Biotechnology Inc., Santa Cruz, CA, USA), anti-MALT1 (B-12, Santa Cruz Biotechnology Inc., Santa Cruz, CA, USA), anti-BCL10 (EP 606Y, Abcam Inc., Cambridge, MA, USA), anti-P-p38 (#9215, Cell Signaling, Danvers, MA, USA), anti-p38 (#9212, Cell Signaling, Danvers, MA, USA), anti-P-IκBα (#9246, Cell Signaling, Danvers, MA, USA), anti-IκBα (sc-371, Santa Cruz Biotechnology Inc., Santa Cruz, CA, USA), anti-P-ERK (#9101, Cell Signaling, Danvers, MA, USA), anti-ERK (#9102, Cell Signaling, Danvers, MA, USA), anti-P-JNK (#9251, Cell Signaling, Danvers, MA, USA), anti-JNK (sc-571, Santa Cruz Biotechnology Inc, Santa Cruz, CA, USA) and anti-actin (MP 6472 J, MP Biochemicals, Santa Ana, CA, USA).

### *In vitro* regulatory T cell differentiation

CD4^+^ T cells were purified from naïve mice using the CD4^+^ T cell isolation kit (Miltenyi Biotech, Bergisch Gladbach, Germany) according to the manufacturer’s instructions. Regulatory T cells were depleted using the CD25 MicroBead kit (Miltenyi Biotech, Bergisch Gladbach, Germany) according to the manufacturer’s instructions. Purified CD4^+^ CD25^-^ T cells were cultured in IMDM supplemented with 5% fetal bovine serum, glutamax, sodium pyruvate, non-essential amino acids and antibiotics in 48-well plates at a density of 0.5 × 10^6^ cells per well. Wells were pre-coated with 5 μg/ml anti-CD3 (clone 145-2C11, BD Biosciences, San Diego, CA, USA) at 37°C for 4 hours and rinsed twice with PBS before applying cells. To induce regulatory T cell differentiation, cells were stimulated with 1 μg/ml anti-CD28 (clone 37.51, BD Biosciences, San Diego, CA), 10 ng/ml hTGFβ (R&D Systems, Minneapolis, MN, USA), 10 μg/ml anti-IFNγ (clone XMG1.2, eBioscience, San Diego, CA, USA) and 10 μg/ml anti-IL-4 (clone 11B11, BD Biosciences, San Diego, CA, USA). Mepazine was omitted or added at a concentration of 5 μM or 10 μM. Cells were analyzed for Foxp3 expression at day 3 and day 5 in culture.

### Flow cytometry

Blood was collected from vehicle and mepazine treated mice at different time points. Red blood cells were removed by two steps of ACK (Lonza, Basel, Switzerland) treatment. Cells were stained with Aqua Live/Dead (Life Technologies), anti-CD16/32 (clone 2.4G2, Fc Block; BD Biosciences, San Diego, CA, USA), anti-CD3-eFluor 450 (clone 145-2C11; eBioscience, San Diego, CA, USA), anti-CD4-FITC (clone GK1.5, BD Biosciences, San Diego, CA, USA), anti-CD25-allophycocyanin-Cy7 (clone PC61; BD Biosciences, San Diego, CA, USA) for 30 minutes at 4°C in PBS supplemented with 0.1% bovine serum albumin (Sigma) and 2 mM EDTA. Prior to staining with anti-Foxp3-PE or anit-Foxp3-APC (clone FJK-16 s), cells were fixed and permeabilized using the Anti-Mouse/Rat Foxp3 Staining Set PE (eBioscience, San Diego, CA, USA) according to the manufacturer’s instructions. Measurements were performed on a BD LSR II cytometer (BD Biosciences, San Diego, CA, USA) and data were analyzed using FlowJo software (Tree Star).

### Statistical analysis

Results are presented as mean ± SEM. Differences between two groups were assessed using a two-tailed Student *t* test or one-way ANOVA. Differences in remission were analyzed using a Gehan-Breslow-Wilcoxon test. Differences were considered significant when *P* <0.05.

## Results

### Mepazine treatment attenuates experimental autoimmune encephalomyelitis induction

**Figure 1:**
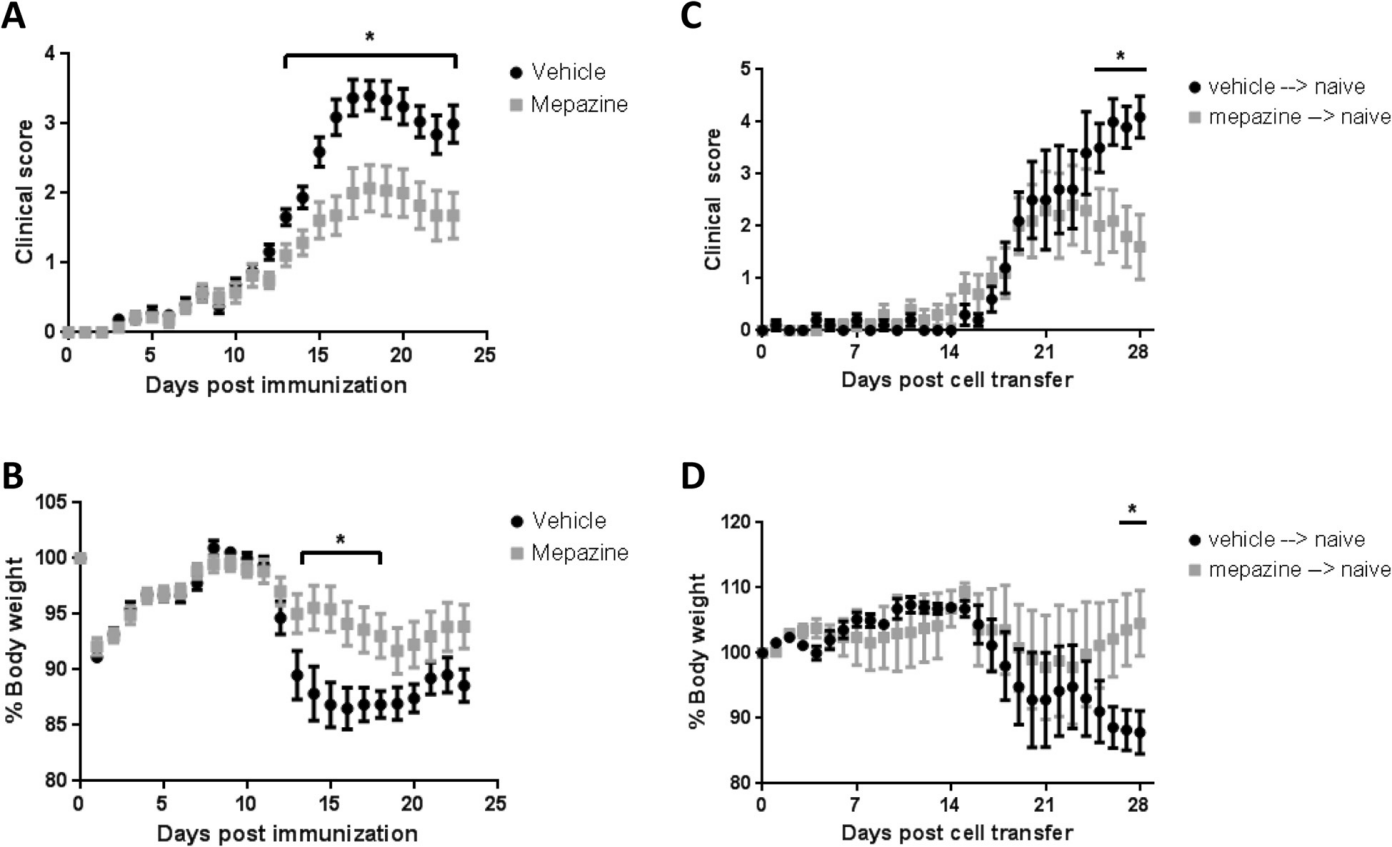
**Treatment with mepazine reduces experimental autoimmune encephalomyelitis (EAE) severity. (A)** Disease progression of mice treated with 8 mg/kg mepazine (n = 14) or vehicle (n = 16) starting at day 7 post-immunization. **(B)** Percentage body weight of mice treated with 8 mg/kg mepazine (n = 14) or vehicle (n = 16) starting at day 7 post-immunization. **(C)** Disease progression of naïve mice receiving splenocytes from MOG_35-55_- immunized, vehicle-treated (n = 5) or mepazine-treated (n = 5) mice. **(D)** Percentage body weight of naïve mice receiving splenocytes from MOG_35-55_-immunized, vehicle-treated (n = 5) or mepazine-treated (n = 5) mice. Data are obtained from two independent experiments and depicted as mean ± SEM *P <0.05.

In order to determine the therapeutic potential of mepazine in EAE, C57BL/6 mice were immunized with MOG_35-55_ peptide and were administered mepazine or vehicle twice daily from day 7 post-immunization onward. Disease progression was evaluated by assessment of clinical scoring and body weight. All vehicle-treated mice developed EAE, following a typical EAE course with onset at approximately day 16 and reaching a maximal clinical score of 3.8 (Figure 1A, Table 1). In contrast, when mice were administered mepazine, disease incidence was reduced to 33% and disease severity was significantly reduced, with mice reaching a maximal clinical score of 2.1 (Figure 1A, Table 1). In addition, whereas vehicle-treated mice lost substantial body weight during EAE, reflecting disease progression, this loss was significantly reduced in mepazine-treated mice (Figure 1B). To determine whether this attenuated EAE was the result of mepazine on the peripheral immune compartment, an adoptive-transfer EAE experiment was performed. Splenocytes from mice immunized with MOG_35-55_ and treated with vehicle or mepazine were re-stimulated *in vitro* with MOG_35-55_ peptide and IL-23, after which they were transferred to naïve mice that were irradiated 1 day prior to cell transfer. Recipient mice receiving cells from vehicle treated mice all developed EAE, with an onset at day 21 and a mean maximal clinical score of 4.4 (Figure 1C, Table 2). In contrast, in recipient mice receiving splenocytes from mepazine-treated mice, disease incidence was reduced to 60%. In addition, the maximal clinical score was reduced to 2.5, suggesting a milder disease progression. Finally, the loss in body weight was also noticeably reduced in recipient mice receiving splenocytes from mepazine-treated donor mice, compared to those receiving splenocytes from vehicle-treated mice (Figure 1D). These results suggest that mepazine alters the auto-reactive peripheral immune response, resulting in a milder EAE induction and progression.

**Table 1:** **Clinical features of MOG**_**35-55**_**-immunized mice treated with mepazine or vehicle**

**Treatment**	**Incidence**	**Day of disease onset**^**a**^	**Mean maximal clinical score**^**a**^
Vehicle	100% (8/8)	16.6 ± 0.9	3.8 ± 0.3
Mepazine	33% (3/10)	16.3 ± 1.3	2.1 ± 0.4^b^

^a^Results are displayed as mean ± SEM.

^b^*P <0.01*.

**Table 2:** **Clinical features of mice receiving donor cells from MOG**_**35-55**_**-immunized, vehicle-treated or mepazine-treated mice**

**Donor cells**	**Incidence**	**Day of disease onset**	**Mean maximal clinical score**
Vehicle	100% (5/5)	21.0 ± 1.5	4.4 ± 0.3
Mepazine	60% (3/5)	17.7 ± 1.3	2.5 ± 0.7

### Mepazine treatment reduces central nervous system inflammation

**Figure 2:**
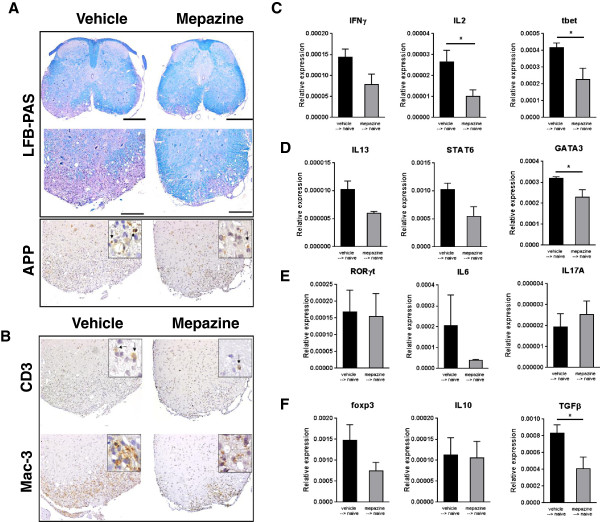
**Reduced central nervous system demyelination, axonal damage and inflammatory cell infiltration and cytokine expression in mepazine-treated mice.** Histopathology on spinal cords of vehicle-treated (n = 5) or mepazine-treated (n = 6) mice. **(A)** Demyelination (blue) and axon damage (APP, brown) were assessed. **(B)** Stainings for infiltrating T cells (CD3, brown) and macrophages (Mac-3, brown) by immunohistochemistry. Scale bars, 500 μm (top panel), 200 μm (lower panel). Expression of Th1 **(C)**, Th2 **(D)**, Th17 **(E)** and Treg-linked **(F)** factors in spinal cord of acceptor mice receiving splenocytes from vehicle- or mepazine-treated mice, 28 days post-splenocyte transfer. Data are depicted as mean ± SEM *P <0.05.

To characterize the milder disease course in mepazine-treated mice, histopathological analysis was performed on spinal cord sections of vehicle- and mepazine-treated mice at day 24 post-immunization. Typically for EAE pathology, vehicle-treated mice demonstrated severe spinal cord demyelination and axonal degeneration, visualized by LFB staining and APP^+^ depositions, respectively (Figure 2A). In addition, inflammatory infiltration of CD3^+^ T cells and Mac-3^+^ macrophages was present in spinal cords of vehicle-treated mice (Figure 2B). In contrast, mepazine-treated mice demonstrated reduced spinal pathology and inflammation (Figure 2A, B, Table 3). Next, we determined the expression of Th1, Th2, Th17 and Treg factors in spinal cord tissue of recipient mice receiving cells from vehicle- or mepazine-treated animals. While most of these factors were clearly increased in vehicle-treated mice during EAE, their expression was suppressed in mepazine-treated conditions (Figure 2C-F). These results demonstrate that mepazine protects mice from EAE pathology by reducing the influx of inflammatory cells, thus attenuating tissue damage in the spinal cord.

**Table 3:** Quantification of spinal cord cell infiltration, demyelination and axonal damage

**Treatment**	**Demyelination (%)**^**a**^	**APP**^**+**^**/mm**^**2a**^	**CD3**^**+**^**/mm**^**2a**^	**Mac-3**^**+**^**/mm**^**2a**^
Vehicle	32.9 ± 6.0	45.4 ± 5.5	114.2 ± 59.2	480.8 ± 191.9
Mepazine	17.6 ± 7.5	29.2 ± 12.9	50.3 ± 22.5	246.5 ± 113.6

Samples were obtained from vehicle (n = 5) or mepazine (n = 6) treated animals. ^a^Results are displayed as mean ± SEM.

### Mepazine abolishes peripheral autoreactive T cell activation

**Figure 3:**
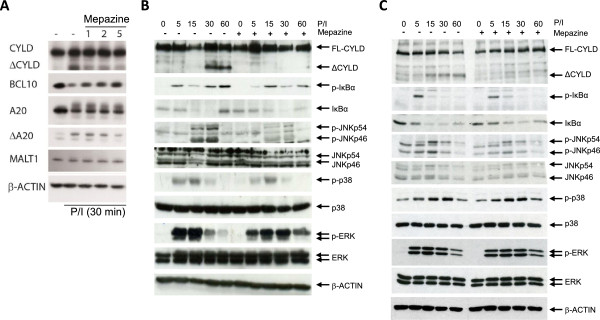
**Mepazine inhibits MALT1 substrate cleavage and reduces NF-κB and JNK activation in T cells. (A-B)** Jurkat T cells and **(C)** purified primary CD4+ T cells were stimulated with PMA plus ionomycin for indicated time points. Where indicated, mepazine was added 90 minutes prior to stimulation. Cleavage of MALT1 substrates CYLD, A20 and BCL10 was analyzed via western blot **(A)**, and lysates were analyzed for IκBα, phospho-IκBα, JNK, phospho-JNK, p38, phospho-p38, ERK, phospho-ERK and actin **(B-C)**.

**Figure 4:**
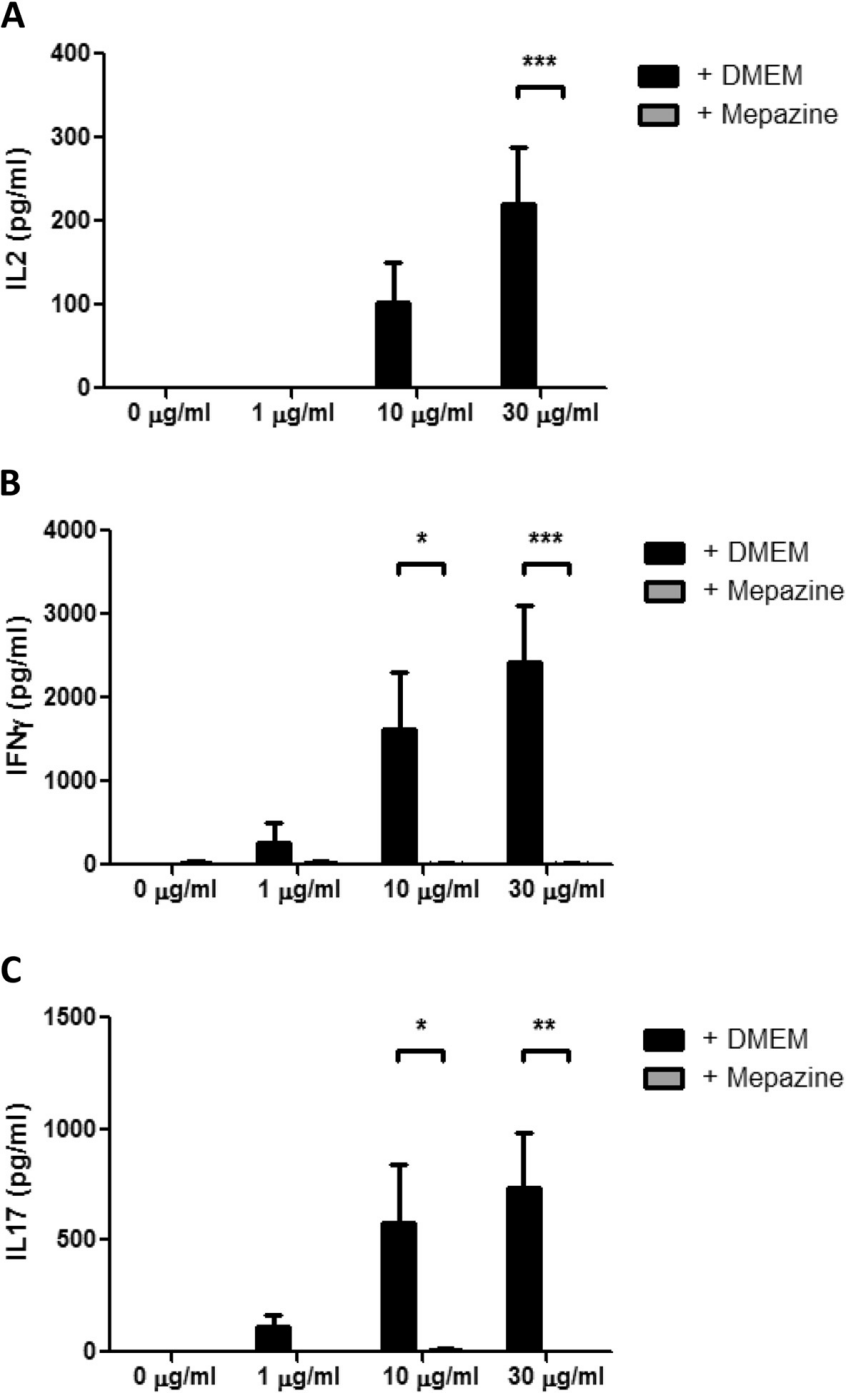
**Peripheral splenocyte activation is inhibited in the presence of mepazine.** Splenocytes from MOG peptide-immunized mice were cultured and stimulated with the indicated concentrations of MOG peptide in the presence or absence of mepazine (n = 4 for each condition). Culture supernatants were collected 48 h after MOG peptide stimulation and assayed for IL-2 **(A)**, IFNγ **(B)** and IL-17 **(C)**. Results are displayed as mean ± SEM. *P <0.05, **P <0.01, ***P <0.001.

Previously, it has been demonstrated that mepazine inhibits T cell activation *in vitro*, likely by acting as an inhibitor of MALT1 protease activity [15]. Similarly, we found that mepazine inhibited the proteolytic processing of A20, BCL-10 and CYLD in PMA/ionomycin stimulated Jurkat T cells (Figure 3A). Furthermore, mepazine strongly inhibited TCR-induced JNK phosphorylation, both in Jurkat cells as in primary T cells (Figure 3B-C). Additionally, a minor delay in TCR-induced NF-κB activation could be shown in Jurkat cells as evidenced by a slightly delayed IκBα phosphorylation and degradation (Figure 3B). To test whether mepazine could also inhibit the activation of autoreactive MOG_35-55_-specific T cells, a T cell recall experiment was performed. Splenocytes from MOG_35-55_-immunized mice were harvested at day 10 post-immunization and re-stimulated with their autoantigen in the presence or absence of mepazine, after which activation of autoreactive T cells was assessed by means of cytokine release in the culture medium. As expected, cells re-stimulated *in vitro* with MOG_35-55_ peptide produced IL-2, IFNγ and IL-17 (Figure 4). In sharp contrast however, the addition of mepazine completely suppressed the production of these cytokines. These results suggest that the protective phenotype of mepazine-treated mice may be due to a reduced activation of autoantigen specific T cells (Figure 4).

### Mepazine treatment does not affect peripheral regulatory T cells

**Figure 5:**
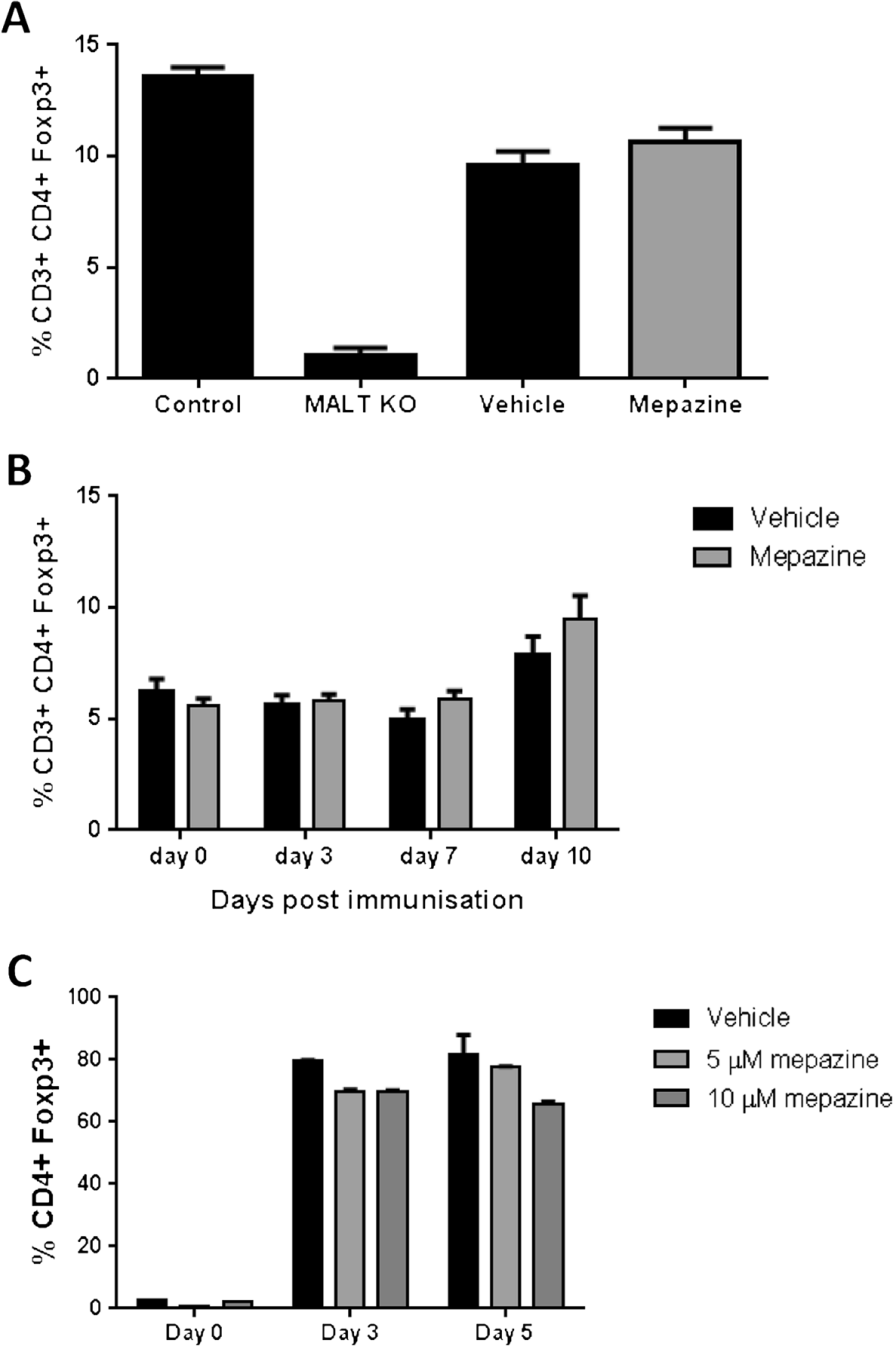
**MALT1 inhibition does not affect regulatory T cell (Treg) development.** Quantification of the percentage CD3+ CD4+ Foxp3+ cells at 24 days post-immunization **(A)** or at different time points after experimental autoimmune encephalomyelitis (EAE) induction **(B)**. Percentage CD3+ CD4+ Foxp3+ cells from control and MALT1 knockout mice are included in panel **(A)**. **(C)** Differentiation of naïve T cells into CD4+ Foxp3+ T cells at different time points *in vitro*. Data are depicted as mean ± SEM.

Mice lacking MALT1 have a severely reduced population of regulatory T cells (Tregs) [17]. To investigate whether treatment with mepazine would have an impact on Treg numbers, splenocytes from MOG_35-55_-immunized and mepazine- or vehicle-treated mice were analyzed at day 24 post-immunization. As previously shown, CD3^+^ CD4^+^ Foxp3^+^ Tregs were largely absent in MALT1 knockouts when compared to control mice; however, the percentage of Tregs in mepazine-treated mice was not significantly reduced compared to control mice, nor did it differ between vehicle- or mepazine-treated animals (Figure 5A). To rule out an effect of mepazine on Treg numbers during the induction phase of EAE, blood was collected at different time points post-immunization and analyzed for Foxp3 expressing T cells. We show that the percentage of Tregs did not differ between vehicle- and mepazine-treated mice (Figure 5B). Finally, the influence of mepazine on Treg differentiation *in vitro* was analyzed, but no effect of mepazine on the capacity of naïve T cells to differentiate into Tregs could be demonstrated (Figure 5C). These results clearly show that mepazine does not influence the Treg population during EAE induction and progression.

### Mepazine suppresses experimental autoimmune encephalomyelitis progression

**Figure 6:**
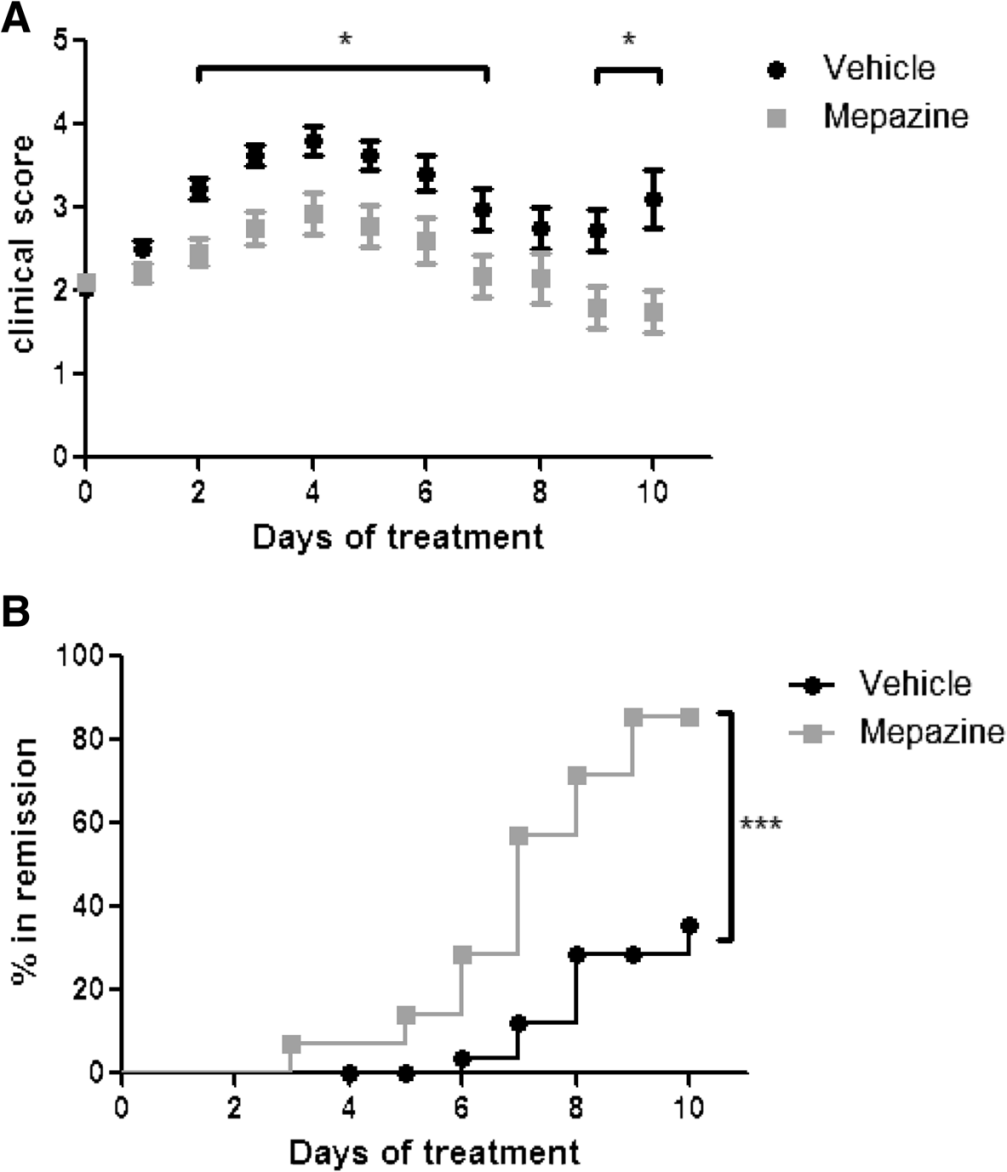
**Treatment with mepazine attenuates experimental autoimmune encephalomyelitis (EAE) and promotes remission. (A)** Clinical scores of mepazine-treated (n = 14) or vehicle-treated (n = 30) mice after treatment starting when mice reach a clinical score of 2.0. Results are obtained from two independent experiments and data are depicted as mean ± SEM. **(B)** Kaplan-Meier plot showing remission (clinical score ≤1.5) after mepazine treatment from mice in figure A. *P <0.05, ***P <0.001.

The above mentioned results suggest that mepazine treatment inhibits EAE by preventing the activation of MOG_35-55_-specific autoreactive T cells. To determine whether mepazine could also be used in a therapeutic setting, we performed an experiment in which mepazine was administered in diseased mice the moment they reach a clinical disease score of 2. Indeed, disease progression was also attenuated under these conditions in mepazine-treated mice when compared to control mice (Figure 6A). In addition, while approximately 40% of vehicle-treated mice went into remission, defined as a clinical score of ≤1.5, this percentage was significantly increased to 80% in mepazine-treated mice (Figure 6B). Together, these results demonstrate that in addition to attenuating the induction of EAE, mepazine can also suppress the progression of the disease.

## Discussion

Recently, the phenothiazine derivative mepazine has been demonstrated to be a potent inhibitor of MALT1 protease activity in ABC-DLBCL and primary T cells. More specifically, mepazine inhibited TCR-induced IL-2 production and proteolytic processing of the MALT1 substrate RelB in primary T cells [15]. In line with this, we found that mepazine prevented the processing of other MALT1 substrates, CYLD, BCL-10 and A20, in primary T cells and Jurkat T cells upon TCR signaling. In addition, mepazine significantly inhibited TCR-induced JNK phosphorylation, consistent with the previously described requirement for MALT1 and MALT1-mediated processing of CYLD for TCR-induced JNK signaling [6]. TCR-induced phosphorylation of p38 and ERK MAP kinases, as well as IκBα phosphorylation, degradation and re-synthesis, were only slightly delayed in mepazine-treated Jurkat cells and not affected in primary T cells, consistent with previous reports showing that MALT1 proteolytic activity is largely dispensable for these events [8].

Given the ability of mepazine to inhibit MALT1 paracaspase activity and TCR-induced signaling, we sought to investigate the *in vivo* therapeutic effect of mepazine during EAE, which is characterized by a T-cell dependent autoimmune response against MOG_35-55_. Mice treated with mepazine prophylactically, before disease symptoms are apparent, are clearly protected in EAE, as shown by a significant reduction in clinical disease symptoms and histopathological parameters such as spinal cord demyelination, axonal degeneration, and inflammatory cell infiltration. These observations likely result from the capacity of mepazine to reduce peripheral autoantigen-specific T cell responses. Similarly, *in vivo* MOG-primed T cells induced a less severe disease course when donor mice were treated with mepazine. Importantly, the EAE inhibitory effect of mepazine was not only observed in a prophylactic setting, but also in a therapeutic setting where mepazine clearly promotes remission.

Previous studies demonstrated that the complete absence of MALT1 in knockout mice leads to a severe defect in the development of Foxp3^+^ Tregs [17], consistent with the essential role of agonist-induced TCR signaling in the expression of Foxp3 and the development of Tregs [19]. Since Tregs have a crucial role in the immune system by preventing autoimmunity [19], limiting immunopathology, and maintaining immune homeostasis, a decrease in Tregs would be harmful in any therapeutic setting that aims to inhibit MALT1 paracaspase activity. Interestingly, no difference in the number of Foxp3^+^ Tregs could be found between vehicle-treated and mepazine-treated mice, demonstrating that a balanced inhibition of TCR signaling by specific inhibition of MALT1 proteolytic activity, leaving its scaffolding function intact, does not affect the development of Tregs and holds promise for the treatment of autoimmune disease.

Mepazine belongs to the class of phenothiazines that are known as first generation antipsychotics and have been in clinical use for the treatment of psychiatric disorders since the early 1950s [20]. Mepazine was in clinical use as an antipsychotic drug under the brand name Pacatal, but two placebo control trials failed to show significant antipsychotic responses in patients leading to market removal in the early 1960s [21]. However, clinical investigations reported no severe adverse effects of mepazine even after long-term treatment for several months at concentrations between 100 to 800 mg q.i.d. [22]. Taking into account that metabolism of phenothiazines in rodents is in general much faster than in humans, these data indicate that mepazine does not cause severe toxicity in humans at concentrations that may be beneficial for MS treatment [23,24]. Certainly, detailed pharmacokinetic (PK) studies comparing mice and humans will be required, but the general ability of phenothiazines to cross the blood-brain barrier further strengthens the concept that mepazine can directly act at the site of the autoimmune lesion [25].

Antipsychotic action of phenothiazines relies on the blockade of the dopamine receptors system, but several reasons reveal that the protective effect of mepazine on EAE is not dependent on the antipsychotic action. In fact, mepazine only insignificantly affects dopamine receptors and this finding corresponds to its clinical ineffectiveness for the treatment of psychiatric disorders [26]. Importantly, we show that EAE is significantly reduced in mice after adoptive transfer of splenocytes from mepazine-treated animals, lending strong support to the model that the compound is acting by inhibiting MALT1 in the autoreactive T cells. Further, from the class of known phenothiazines mepazine is clearly the most effective MALT1 inhibitor [15]**,** and the allosteric mode of MALT1 inhibition has been confirmed by mutation of the mepazine binding site on MALT1 [16]. We cannot exclude that other MALT1 independent processes may contribute to the EAE protection, but the effective inhibition of MALT1 in T cells, the impaired T cell responses, the reduced CNS inflammation and the decreased disease incidence after adoptive splenocyte transfer from mepazine-treated mice clearly point out that MALT1 is the major target of mepazine in this animal model.

## Conclusions

MALT1 targeting with mepazine and other small compound inhibitors has recently been shown to hold significant potential in the treatment of lymphoma. Our studies now demonstrate the efficacy of MALT1 protease targeting in the treatment of autoimmune disease. The present study illustrates its efficiency in a murine model of MS and it will be interesting to study the effect of MALT1 targeting in other inflammatory diseases such as rheumatoid arthritis, psoriasis and inflammatory bowel disease that involve increased T cell receptor signaling. Even though more effective MALT1 inhibitors may be available in the future, the previous clinical use may facilitate a repurposing of mepazine and foster the translation of our findings to the treatment of severe autoimmune diseases in the clinic.

## Abbreviations

ABC-DLBCL: activated B cell-like diffuse large B cell lymphoma; APP: amyloid precursor protein; BBB: blood-brain barrier; CNS: central nervous system; CYLD: cylindromatosis; EAE: experimental autoimmune encephalomyelitis; i.p.: intraperitoneally; i.v.: intravenously; H & E: hematoxylin and eosin; LFB: Luxol fast blue; MALT1: mucosa-associated lymphoid tissue 1; MOG: myelin oligodendrocyte glycoprotein; MS: multiple sclerosis; NF-κB: nuclear factor-κB; TCR: T cell receptor; Tregs: regulatory T cells.

## Competing interests

The authors declare that they have no competing financial interests.

## Authors’ contributions

CMG, RB and GVL designed the study; CMG, LE, PW, JS, SV, AD and DN performed experiments; CMG, DK, MP, RB and GVL analyzed data. CMG, DK, RB and GVL wrote the manuscript. All authors read and approved the final manuscript.

## Authors’ information

RB and GvL share senior authorship.
